# Berberine ameliorates cartilage degeneration in interleukin-1β-stimulated rat chondrocytes and in a rat model of osteoarthritis *via* Akt signalling

**DOI:** 10.1111/jcmm.12186

**Published:** 2013-11-28

**Authors:** Honghai Zhao, Tongen Zhang, Chun Xia, Lei Shi, Shaojie Wang, Xinpeng Zheng, Tianhui Hu, Bing Zhang

**Affiliations:** a Zhongshan Hospital, University of XiamenXiamen, Fujian, China; b School of Medicine, University of XiamenXiamen, Fujian, China

**Keywords:** Akt, berberine, cartilage degeneration, IL-1β-stimulated chondrocytes, rat OA model

## Abstract

Berberine, a plant alkaloid used in Chinese medicine, has broad cell-protective functions in a variety of cell lines. Chondrocyte apoptosis contributes to the pathogenesis of cartilage degeneration in osteoarthritis (OA). However, little is known about the effect and underlying mechanism of berberine on OA chondrocytes. Here, we assessed the effects of berberine on cartilage degeneration in interleukin-1β (IL-1β)-stimulated rat chondrocytes and in a rat model of OA. The results of an MTT assay and western blotting analysis showed that berberine attenuated the inhibitory effect of IL-1β on the cell viability and proliferating cell nuclear antigen expression in rat chondrocytes. Furthermore, berberine activated Akt, which triggered p70S6K/S6 pathway and up-regulated the levels of aggrecan and Col II expression in IL-1β-stimulated rat chondrocytes. In addition, berberine increased the level of proteoglycans in cartilage matrix and the thickness of articular cartilage, with the elevated levels of Col II, p-Akt and p-S6 expression in a rat OA model, as demonstrated by histopathological and immunohistochemistry techniques. The data thus strongly suggest that berberine may ameliorate cartilage degeneration from OA by promoting cell survival and matrix production of chondrocytes, which was partly attributed to the activation of Akt in IL-1β-stimulated articular chondrocytes and in a rat OA model. The resultant chondroprotective effects indicate that berberine merits consideration as a therapeutic agent in OA.

## Introduction

One of the main symptoms in osteoarthritis (OA) is the progressive degeneration of articular cartilage including chondrocyte loss and degradation of the extracellular matrix (ECM) 1. Chondrocyte apoptosis that is higher in human OA cartilage could be involved in the initiation and severity of articular cartilage degeneration 2,3. Therefore, it is a potential approach to generate effective and durable treatments against apoptosis of OA chondrocytes 4–6. Proinflammatory cytokines secreted by chondrocytes, such as interleukin-1β (IL-1β) and tumour necrosis factor-α, contribute to the progression of OA 7–9. Specifically, IL-1β can trigger chondrocyte apoptosis and is commonly used as a modulating and chondrocyte apoptosis-inducing agent 10–12. The chondrocytes treated with IL-1β thus mimicked OA chondrocytes 11,12.

Berberine, a botanical alkaloid present in the root and bark of several plants, is the major active component of rhizoma coptidis. Previous reports have shown that berberine has multiple pharmacological effects including antibiotic, anticancer, and anti-inflammatory 13,14. It is recently concerned about the therapeutic effect of berberine on OA 15,16. Berberine decreased IL-1β-induced proteoglycan release and nitric oxide production in IL-1β-induced rat articular chondrocytes 15. Berberine blocked the release of collagen and proteoglycan from IL-1β-stimulated rabbit cartilage and down-regulated matrix metalloproteinases (MMPs) in rabbit chondrocytes 16. However, the underlying mechanism of berberine remains unclear.

The protein kinase B (Akt) could be phosphorylated and activated by extracellular factors in a phosphatidylinositol 3-kinase (PI3K)-dependent fashion, participating in a variety of important physiological functions. Specifically, activated PI3K/Akt pathway was involved in regulating chondrocyte survival 17–19. Increased expression of the Akt inhibitor TRB3 in OA chondrocytes inhibited IGF-1-mediated cell survival and proteoglycan synthesis 18. IGF-I stimulation of glycosaminoglycan synthesis by chondrocytes required the activation of PI3K/Akt pathway 19. Our previous results also indicated that 17β-edtradiol and nicotine promoted cell survival *via* activating PI3K/Akt pathway in IL-1β-stimulated rat chondrocytes 6,20. Activated PI3K/Akt pathway is therefore involved in OA progression.

The purpose of this study is to investigate the effect of berberine on IL-1β-stimulated rat chondrocytes, as well as articular cartilage in a rat OA model, and to elucidate the underlying mechanism associated with Akt signalling.

## Materials and methods

### Reagents

Berberine (purity ≥98%) was purchased from Sigma-Aldrich (Shanghai, China), dissolving in double distilled water for stock preparation. The required concentrations of berberine for individual experiments were made by further dilution of the stock preparation with culture medium when needed. Recombinant rat IL-1β was from PeproTech (Rocky Hill, NJ, USA). Antibodies against Akt, p-Akt-Ser473, p-p70S6K-Thr389, p-S6-Ser235/236, proliferating cell nuclear antigen (PCNA), aggrecan, Col II, GAPDH, and PI3K inhibitor (LY294002) were purchased from Cell Signaling Technology Inc. (Beverly, MA, USA) and Santa Cruz Biotechnology (Santa Cruz, CA, USA) respectively. Other reagents were of the highest grade commercially available.

### Isolation and culture of rat chondrocytes

Neonatal male Sprague–Dawley rats (within 24 hrs after birth) were killed after approval of the ethical Committee of Medical School, Xiamen University (ID No. 20110920), and articular cartilages were removed under sterile conditions (Fig. S1). Rat articular chondrocytes were cultured as previously described 6,21.

### Stimulation with IL-1β and treatment with berberine

Adherent rat chondrocytes at 60–70% confluency were cultured with serum-starved medium (DMEM/F12 supplemented with 1% FBS) for 12 hrs, and then stimulated with IL-1β (10 ng/ml) for 2 hrs (Fig. S1). Cells were then treated with indicated concentrations of berberine in the presence or absence of LY294002 and harvested at different times as required to be subjected to different experimental procedures.

### Cell viability analysis

The cell viability was detected using 3-(4,-Dimethylthiazol-2-y)-2,5-diphenyl-tetrazolium bromide (MTT) assay as described previously 6.

### Protein extraction and western blotting analysis

Cells collected by centrifugation were lysed as previously described 9. Protein extracts were electrophoresed on 8–12% denaturing gel and transferred to PVDF membrane (GE Healthcare, Fribourg, Switzerland) for western blotting analysis 20. The signal was detected using a chemiluminescent detection system according to the manufacturer's instructions (Pierce, Rockford, IL, USA).

### Establishment of a rat OA model

This study was carried out in strict accordance with the recommendations in the Guide for the Care and Use of Laboratory Animals of the National Institutes of Health. The protocol was approved by the Committee on the Ethics of Animal Experiments of the University of Xiamen (ID No. 20110920). Nine-week-old male Sprague–Dawley rats (250–300 g) were used in the following experiments. The animals were acclimatized to the laboratory environment for 1 week before the experiments. Rats were randomly divided into six groups (*n* = 6) including Sham-operated group, OA-induction, OA+ the vehicle (water), OA+Berberine (Low-dose), OA+Berberine (Middle-dose), and OA+Berberine (High-dose). The right knee joint of each rat was the experimental joint. The OA model was induced with anterior cruciate ligament transection in combination with resection of medial menisci (ACLT + MMx) as previously described 22. After surgery, the joint surface was washed with sterile saline solution, and both capsule and skin were sutured. From the 4th week after surgery, different doses of berberine [low-dose (7 μg/kg), middle-dose (14 μg/kg), high-dose (28 μg/kg)] and the vehicle (water) were injected into the knee joints, and rats were not killed until the 10th week after surgery.

### Histopathological scores

Semi-quantitative histopathological grading was performed according to a modified Mankin scoring system established for grading OA changes 23–25. Mankin scores normally consider five characteristics: structure, chondrocyte number, chondrocyte clustering, proteoglycan content, and subchondral bone plate and/or tidemark change 26. Three sections from each sample 100 μm apart were scored by two different blinded observers, for a maximum possible score of 14. Low scores are consistent with minor degenerative cartilaginous lesions, whereas high total scores are indicative of more pronounced cartilaginous changes.

Glycosaminoglycan was stained red by Safarinin-O, and the total and red-stained areas in the articular cartilage of each proximal tibia were measured using Image-Pro Plus 6.0 software. The ratio of red-stained area to total area (red/total) in each group was used to evaluate the effect of berberine on ECM of chondrocytes.

### Immunohistochemistry technique

As described in the manufacturer's instructions (MAIXIN.BIO, Fuzhou, China), the tibia articular sections of paraffin-embedded knees were incubated overnight at 4°C with primary antibody: Col II (1:200), p-S6 (1:400), and p-Akt (1:100 dilutions) and subsequently, with secondary antibody (1:400) for 60 min. Diaminobenzidine (DAB) was used to visualize the immunohistochemical reaction followed by being counterstained with haematoxylin. Finally, dark brown cells were considered to be positive. Photomicrographs were taken with OLYMPUS BX41 microscope equipped with a digital camera. The relative density of immunostaining (density/area) was measured using Image-Pro Plus 6.0 Software 27.

### Statistical analysis

Data were expressed as mean ± SEM for at least three separate determinations for each group. The differences between the groups were examined for statistical significance using the Student's *t*-test and one-way anova with SPSS software. A value of *P* < 0.05 was considered as being significant.

## Results

### Berberine promotes cell survival through activating Akt signalling in IL-1β-stimulated rat chondrocytes

**Fig 1:**
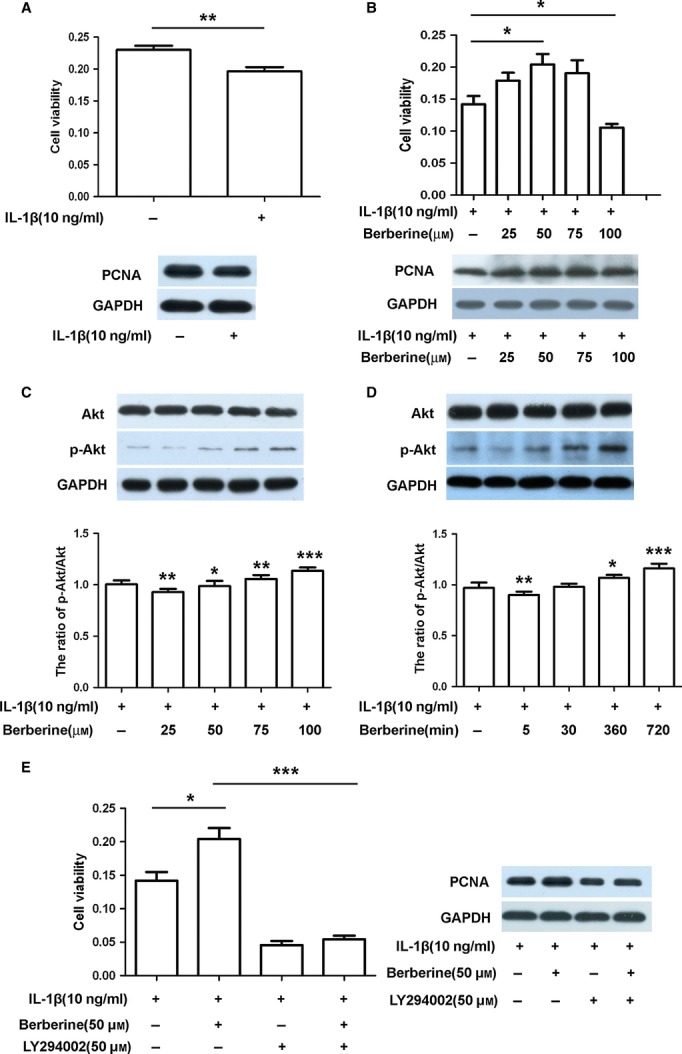
Berberine promotes cell survival through activating Akt signalling in IL-1β-stimulated rat chondrocytes. (A) Cells were treated with IL-1β (10 ng/ml) for 2 hrs, and the cell viability and proliferating cell nuclear antigen (PCNA) expression were measured by the MTT assay and western blotting respectively. (B) Cells were pre-treated with IL-1β (10 ng/ml) for 2 hrs prior to treatment with different concentrations of berberine (25, 50, 75 and 100 μM) for 24 hrs, and the cell viability and PCNA expression were measured by the MTT assay and western blotting respectively. (C) Cells were pre-treated with IL-1β (10 ng/ml) for 2 hrs prior to treatment with different concentrations of berberine (25, 50, 75 and 100 μM) for 12 hrs, and the protein expression of Akt and p-Akt was detected by western blotting using rat anti-Akt, p-Akt and GAPDH antibodies. (D) Cells were pre-treated with IL-1β (10 ng/ml) for 2 hrs prior to treatment with berberine (50 μM) for the indicated time, and the protein expression of Akt and p-Akt was detected by western blotting analysis using rat anti-Akt, p-Akt and GAPDH antibodies. (E) Cells were pre-treated with IL-1β (10 ng/ml) for 2 hrs prior to treatment with or without LY294002(50 μM) for 1 hr, followed by berberine (50 μM) for 12 hrs, and the cell viability and PCNA expression were measured by the MTT assay and western blotting respectively. The blots were normalized to an endogenous protein (GAPDH).The values represent the mean ± SEM of three to five independent experiments, each yielding similar results (*P < 0.05,***P < 0.001).

To investigate the effect of IL-1β on rat chondrocytes, cells were treated by IL-1β (10 ng/ml) for 2 hrs. The cell viability was reduced compared with the untreated group, as determined by an MTT assay (upper panel, Fig. 1A, *P < 0.05). Meanwhile, the expression of PCNA was suppressed in IL-1β-stimulated rat chondrocytes using western blotting analysis (lower panel, Fig. 1A). Thus, IL-1β could inhibit the proliferation of rat chondrocytes. Subsequently, the effect of berberine on IL-1β-stimulated rat chondrocytes was evaluated. As shown in Figure 1B, although 100 μM berberine caused the decrease in cell viability, 50 μM berberine enhanced the cell viability of IL-1β-stimulated chondrocytes significantly (upper panel, Fig. 1B, *P < 0.05, *versus* untreated group). The addition of berberine also enhanced the level of PCNA expression in IL-1β-stimulated chondrocytes (lower panel, Fig. 1B). The data demonstrate that definite concentration of berberine could attenuate the inhibitory effect of IL-1β on cell viability and PCNA expression.

Whether Akt signalling was involved in the effect of berberine on IL-1β-stimulated rat chondrocytes is not known. Protein levels of p-Akt and Akt, therefore, were assessed in the presence or absence of berberine. The result of western blotting analysis showed that different concentrations and treating times of berberine did not change the total Akt level in IL-1β-stimulated rat chondrocytes (Fig. 1C and D). The higher concentrations of berberine markedly up-regulated p-Akt expression, although 25 μM berberine down-regulated p-Akt expression (Fig. 1C, *P < 0.05, **P < 0.01, ***P < 0.001, *versus* untreated group). When IL-1β-stimulated rat chondrocytes exposed to berberine (50 μM) treatment for the longer times (360 min. and 720 min.), p-Akt expression was up-regulated significantly (Fig 1D, *P < 0.05, **P < 0.01, ***P < 0.001, *versus* untreated group). Berberine thus activated Akt in IL-1β-stimulated rat chondrocytes in a concentration and time-dependent manner. In addition, the addition of LY294002 (50 μM), the inhibitor of PI3K, reduced the berberine-mediated increase in cell viability and PCNA expression in IL-1β-stimulated rat chondrocytes (Fig. 1E, ***P < 0.001, *versus* those treated with berberine). Therefore, definite concentration of berberine promotes cell survival through activating Akt signalling in IL-1β-stimulated rat chondrocytes.

### Activated Akt by berberine triggers p70S6K/S6 pathway in IL-1β-stimulated rat chondrocytes

**Fig 2:**
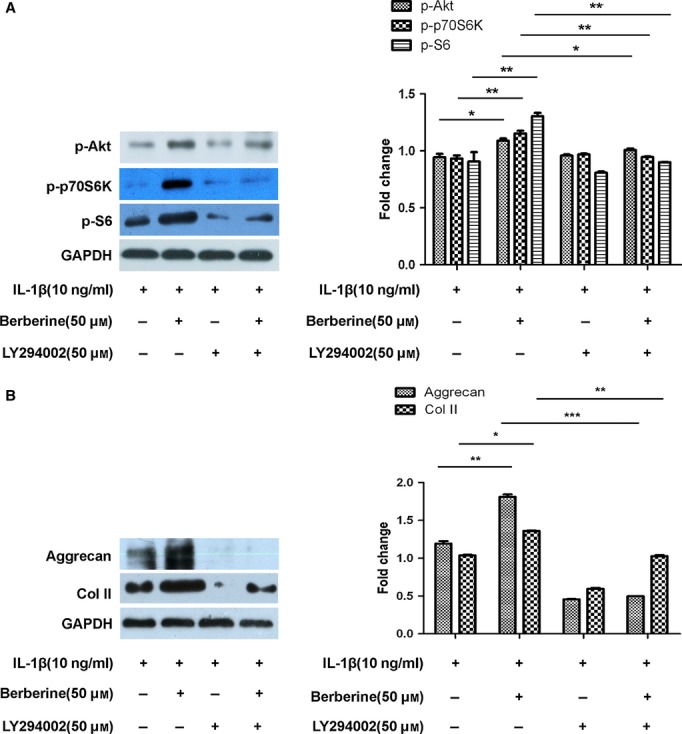
Berberine enhances protein synthesis *via* Akt/p70S6K/S6 pathway in IL-1β-stimulated rat chondrocytes. Cells were pre-treated with IL-1β (10 ng/ml) for 2 hrs prior to treatment with or without LY294002 (50 μM) for 1 hr, followed by berberine (50 μM) for 12 hrs. (A) The protein expression of p-Akt, p-p70S6K, and p-S6 was detected by western blotting using rat anti-p-Akt, p-p70S6K, p-S6 and GAPDH antibodies. (B) The protein expression of aggrecan, and Col II was detected by western blotting using rat anti-proliferating cell nuclear antigen, aggrecan, Col II and GAPDH antibodies. The blots were normalized to an endogenous protein (GAPDH). The values represent the mean ± SEM of three to five independent experiments, each yielding similar results (*P < 0.05,**P < 0.01, ***P < 0.001).

Akt has been reported to modulate protein synthesis through phosphorylation of its substrates such as p70S6K, and S6. The phosphorylation level of p70S6K and S6 in IL-1β-stimulated rat chondrocytes, therefore, was evaluated with western blotting when cells were treated with LY294002 (50 μM) for 1 hr prior to treatment with 50 μM berberine for 12 hrs. Berberine increased the levels of p-p70S6K and p-S6 expression as well as p-Akt (Fig. 2A, *P < 0.05, *^*^P < 0.01, *versus* untreated group). The addition of LY294002 attenuated the berberine-mediated increase in p-p70S6K and p-S6 expression as well as p-Akt (Fig. 2A, *P < 0.05, **P < 0.01, *versus* those treated with berberine). Because the loss of aggrecan and collagenous network is the primary event leading to the destruction of cartilage, we then detected the levels of aggrecan and Col II expression in IL-1β-stimulated rat chondrocytes. As shown in Figure 2B, the levels of aggrecan and Col II expression appeared to be up-regulated significantly by berberine (Fig. 2B, *P < 0.05, ***P < 0.001, *versus* untreated group). The addition of LY294002 attenuated the berberine-mediated increase in aggrecan and Col II expression (Fig. 2B, **P < 0.01, ***P < 0.001, *versus* treated with berberine), indicating that berberine might modulate matrix production *via* activating Akt signalling. Taken together, activated Akt by berberine triggered p70S6K/S6 pathway and modulated matrix production in IL-1β-stimulated rat chondrocytes.

### Berberine ameliorates the cartilage damage in a rat model of OA with the increase in p-Akt and p-S6 expression

**Fig 3:**
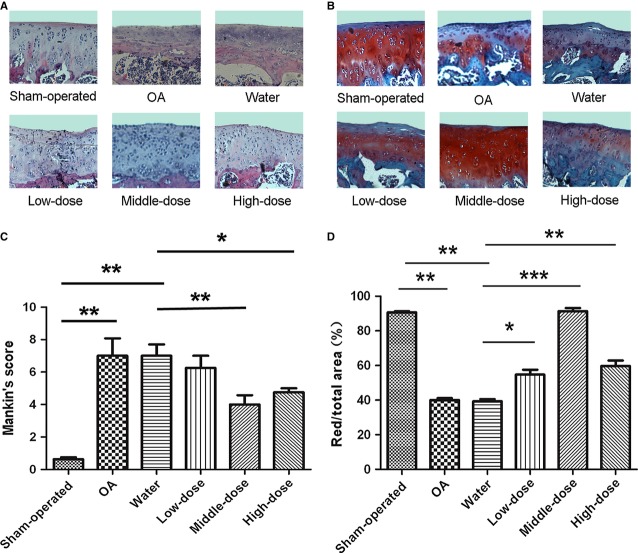
Berberine repairs cartilage damage in a rat osteoarthritis model. Specimens were longitudinally cut into 3–5 μm sections. (A) Specimens in different treated groups were examined using haematoxylin and eosin staining (original magnification ×200). (B) Specimens in different treated groups were detected using Safranin-O fast green staining (original magnification ×200). (C) Histopathological scores were performed by Mankin's score. (D) The index of matrix production was evaluated by the percentage of red area/total area. The values represent the mean ± SEM of three to five independent experiments, each yielding similar results (*P < 0.05, **P < 0.01, ***P < 0.001).

Articular cartilage in sham joint group possesses regular morphological structure (Fig. S2A and Fig. 3A). OA-induction group with ACLT + MMx exhibited evidently the reduction in chondrocytes and articular cartilage thickness with the irregular morphological structure (Fig. S2B and S3; Fig. 3A). Water-injected OA-induction group is similar to OA-induction group, indicating that the injection with water had no influence on OA morphological structure (Fig. S2C and S3; Fig. 3A). Intra-articular injection with different doses of berberine all led to the increase in articular cartilage thickness significantly and the amelioration of cartilage damage, compared with OA-induction and water-injected OA-induction groups (Fig. S2D–F and S3; Fig. 3A). Specifically, more superficial chondrocytes were noted in articular cartilage from middle-dose and high-dose berberine-treated groups (Fig. 3A). The severity of cartilage destruction was then scaled using Mankin's method. The severity of OA-induction group (7.0 ± 0.52) and water-injected OA-induction group (7.1 ± 1.10) was much higher than that of the sham-operated group (0.6 ± 0.80; Fig. 3C, **P < 0.01), indicating the reliability of our OA model and the lack of a placebo effect in this model. The severity of middle-dose berberine-injected OA-induction group (4.0 ± 0.70) and high-dose berberine-injected OA-induction group (4.8 ± 1.12) was lower than that of water-injected OA-induction group (7.1 ± 1.10; Fig. 3C, *P < 0.05, **P < 0.01), excluding the low-dose berberine-injected OA group (6.3 ± 0.75). It is suggested that intra-articular with the definite doses of berberine, especially the middle-dose berberine, might have evident protective effect on the cartilage damage in rat OA model.

**Fig 4:**
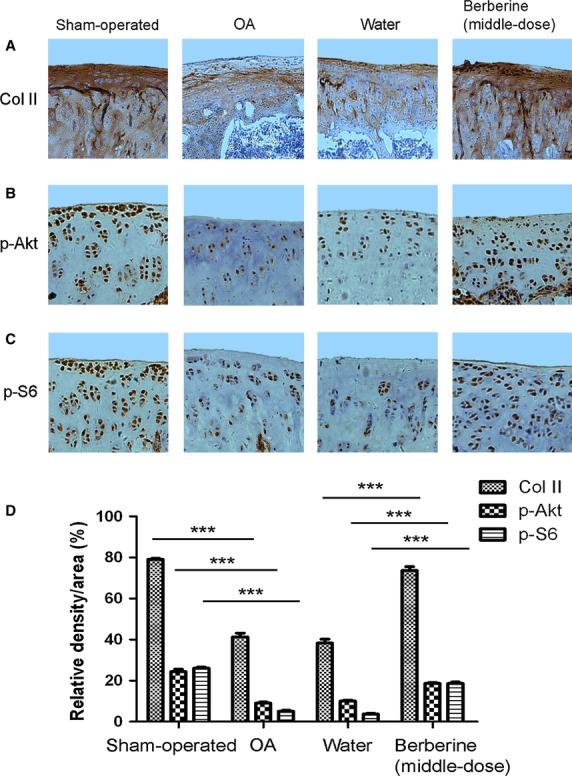
Berberine enhances the levels of Col II, p-Akt and p-S6 expression in a rat osteoarthritis model. Specimens were longitudinally cut into 3-μm sections and the levels of Col II (A), p-Akt (B) and p-S6 (C) expression in different treated groups were detected by immunohistochemisty technique (A, original magnification ×100; B and C, original magnification ×200). (D) Histogram of the relative density of OD of chondrocytes. The values represent the mean ± SEM of three to five independent experiments, each yielding similar results (***P < 0.001).

Concomitantly, Safranin-O staining for proteoglycans showed a distinct reduction in the cartilage matrix in OA-induction and water-injected OA-induction groups by the ratio of red-stained area to total area (red/total), indicating the matrix degradation of OA (Fig. 3B and D, **P < 0.01, *versus* sham-operated group). The matrix degradation in berberine-injected OA-induction groups was less than water-injected OA-induction group (Fig. 3B and D, *P < 0.05, **P < 0.01, ***P < 0.001). It is consistent with the increase in Col II expression in middle-dose berberine-injected group, as determined by the immunohistochemistry technique (Fig. 4, ***P < 0.001, *versus* water-injected group). These data indicated that berberine promoted matrix production in a rat model of OA.

In addition, the levels of p-Akt and p-S6 expression declined significantly in OA-induction group (Fig. 4, ***P < 0.001, *versus* sham-operated group), and enhanced in middle-dose berberine-injected group (Fig. 4, ***P < 0.001, *versus* water-injected group). Therefore, intra-articular injection with definite doses of berberine, in part, ameliorated the cartilage damage with the elevated levels of p-Akt and p-S6 expression.

## Discussion

In this study, we found that berberine promoted chondrocyte survival and matrix production in IL-1β-stimulated articular chondrocytes, one type of model OA chondrocytes, and in a rat model of OA. Specifically, berberine could activate Akt and the underlying mechanism of berberine is associated with Akt/p70S6K/S6 pathway.

Increasing chondrocyte apoptosis is considered a key pathological feature of OA, and anti-apoptosis is a novel therapeutic target for OA 4–6. Some evidence showed that berberine had anti-apoptosis properties in some cell lines. For example, berberine protected against mesenchymal stem cells apoptosis 28. Berberine pre-treatment promoted PC12 cells survival and inhibited apoptosis under hypoxia condition 29. In this study, definite concentrations of berberine could also enhance the cell viability and the level of PCNA expression in IL-1β-stimulated chondrocytes, exhibiting its anti-apoptosis effect on OA chondrocytes similar to previous studies 28–31. Furthermore, we observed that berberine up-regulated the levels of aggrecan and Col II expression in IL-1β-stimulated rat chondrocytes and in a rat OA model. These data, together with evidence indicating that berberine decreased the levels of MMP-1, MMP-3 and MMP-13 expression and increased TIMP-1 at the mRNA and protein levels in an experimental rat OA model 15, and that berberine blocked the release of collagen and proteoglycan from IL-1β-stimulated rabbit cartilage and down-regulated MMPs in rabbit chondrocytes 16 strongly suggest that berberine promoted matrix production in IL-1β-stimulated rat chondrocytes and in a rat OA model. Therefore, berberine has a chondroprotective effect on OA chondrocytes, exhibiting the maintenance of cell survival and promotion of matrix production, and may represent a therapeutic potential for the treatment of cartilage damage in OA.

In this study, it is worth mentioning that berberine activated Akt in IL-1β-stimulated rat chondrocytes and in a rat OA model. A role for Akt in berberine-stimulated cell lines is previously demonstrated by the data indicating that berberine inhibited the metastatic potential of breast cancer cells *via* Akt pathway modulation 32, and that berberine might induce autophagic cell death in HepG2 and MHCC97-L cells through activation of Beclin-1 and inhibition of the mTOR-signalling pathway by suppressing the activity of Akt and up-regulating p38 MAPK signalling 33. Therefore, it is certain that activated PI3K/Akt by berberine was involved in the chondroprotective effect of berberine in IL-1β-stimulated chondrocytes and in a rat OA model. The precise mechanisms by which Akt regulates this process are not fully understood. More importantly, accumulating evidence now show that S6 possesses the RxRxxS/T motif, which can be phosphorylated by AGC kinase family members such as Akt and RSK, indicating that PI3K/Akt mediated phosphorylation of S6 34,35. Our results thus add important information towards a full understanding of the contribution of activating Akt to the chondroprotective effect of berberine by identifying p70S6K/S6 as a downstream cascade that Akt can regulate protein synthesis.

In addition, we observed that Akt activity was also required for berberine-induced elevation of aggrecan and Col II expression levels in IL-1β-stimulated rat chondrocytes and in a rat OA model. It is consistent with previous findings that the expression of constitutively active Akt in human articular chondrocytes resulted in significant increases in proteoglycan synthesis, Col II synthesis and expression, as well as Sox9 expression 36, and that the inhibition of PI3K/Akt signalling pathway has been shown to inhibit chondrocyte proteoglycan synthesis and reduce chondrocyte survival 18. Therefore, berberine-induced Akt activation and triggered p70S6K/S6 pathway, leading to the promotion of protein synthesis, cell survival and matrix production in rat IL-1β-stimulated chondrocytes and in a rat OA model.

As it is often difficult to extrapolate *in vitro* data to a clinical setting, the animal OA model was frequently induced to imitate the complexity with which biological factors and cytokines work together in the metabolic system to maintain or disrupt homeostasis 37,26,38. In our rat OA model with ACLT+MMx *in vivo*, the Mankin's scores in OA-induction and water-injected OA-induction groups were lower than that in the berberine-injected groups (middle-dose and high-dose group), and more chondrocytes, thicker cartilage layer and higher matrix production were observed in berberine-injected groups, demonstrating the chondroprotection of berberine on the articular cartilage from OA. In addition, the chondroprotection of berberine is associated with Akt and S6 phosphorylation, consistent with the observation in IL-1β-stimulated rat chondrocytes *in vitro*. Berberine therefore has a potential therapeutic ability for cartilage degeneration in a rat model of OA. Hu *et al*. have also reported that berberine exerted a chondroprotective effect in a rat OA model 15.

In conclusion, this study demonstrated for the first time that definite concentration of berberine protected articular cartilage from damage of OA *via* activating Akt/p70S6K/S6 signalling pathway, promoting cell survival and matrix production in IL-1β-stimulated rat articular chondrocytes and in a rat OA model. The resultant chondroprotective effects indicate that berberine merits consideration as a therapeutic agent in OA.

## Supporting information

Figure S1 The morphological observation of the cultured rat chondrocytes and IL-1β-stimulated chondrocytes.

Figure S2 The gross anatomical change in the cartilage damage in a rat OA model injected by berberine. (A)Sham-operated group. (B) OAinduction group. (C) OA+the vehicle (water) group. (D) OA+Berberine (Low-dose) group. (E)OA+Berberine (Middle-dose). (F) OA+Berberine (High-dose).

Figure S3 Histogram of the relative thickness of articular cartilage in a rat OA model injected by berberine.
