# Supplementary figures and images for: Berberine ameliorates cartilage degeneration in interleukin-1β-stimulated rat chondrocytes and in a rat model of osteoarthritis via Akt signalling
Source: J Cell Mol Med. 2013 Nov 28;18(2):283–92. doi: 10.1111/jcmm.12186 (PMC3930415; doi:10.1111/jcmm.12186)

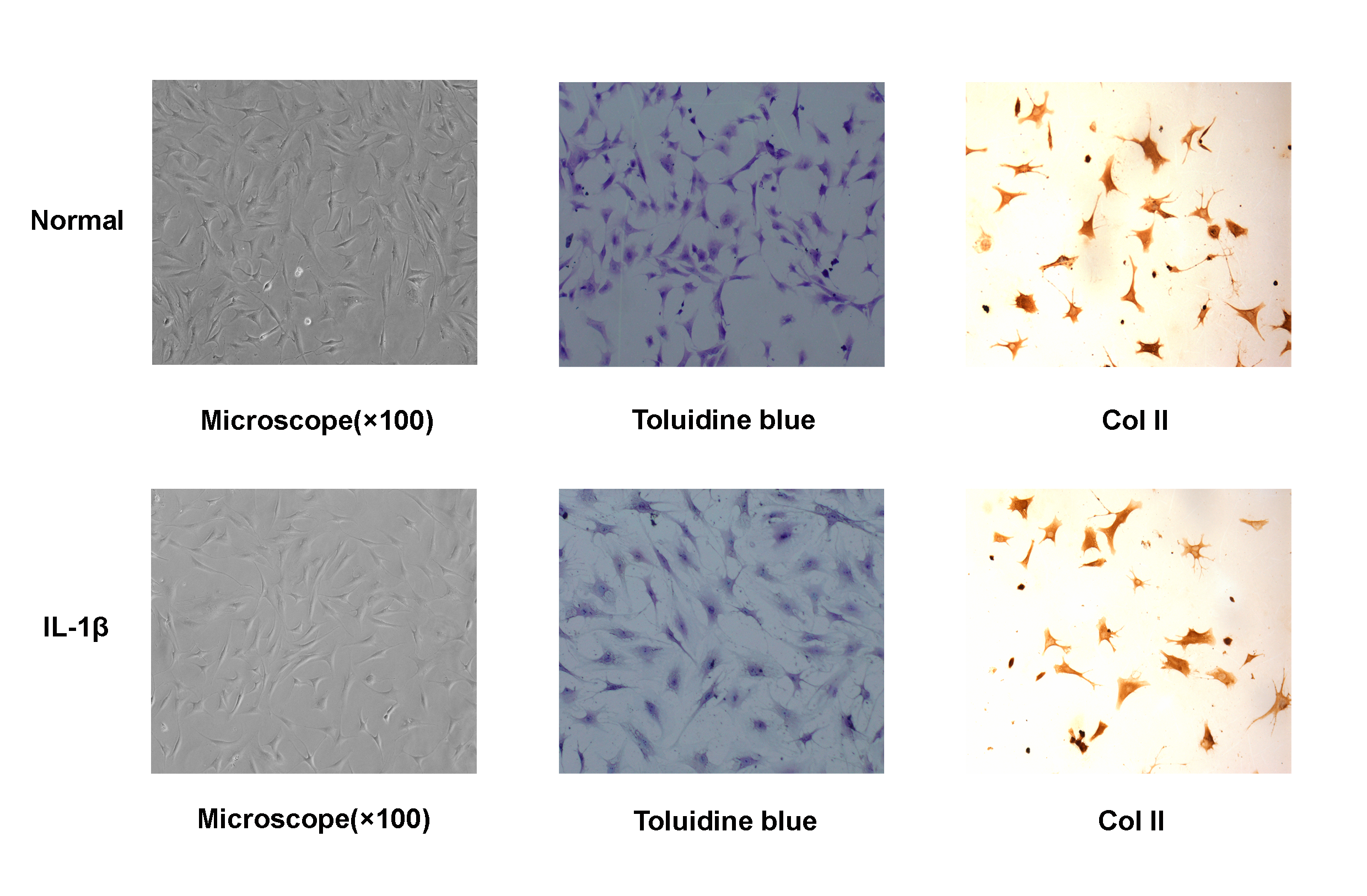

Supplement: Figure S1 — The morphological observation of the cultured rat chondrocytes and IL-1β-stimulated chondrocytes. [file jcmm0018-0283-sd1.tif]

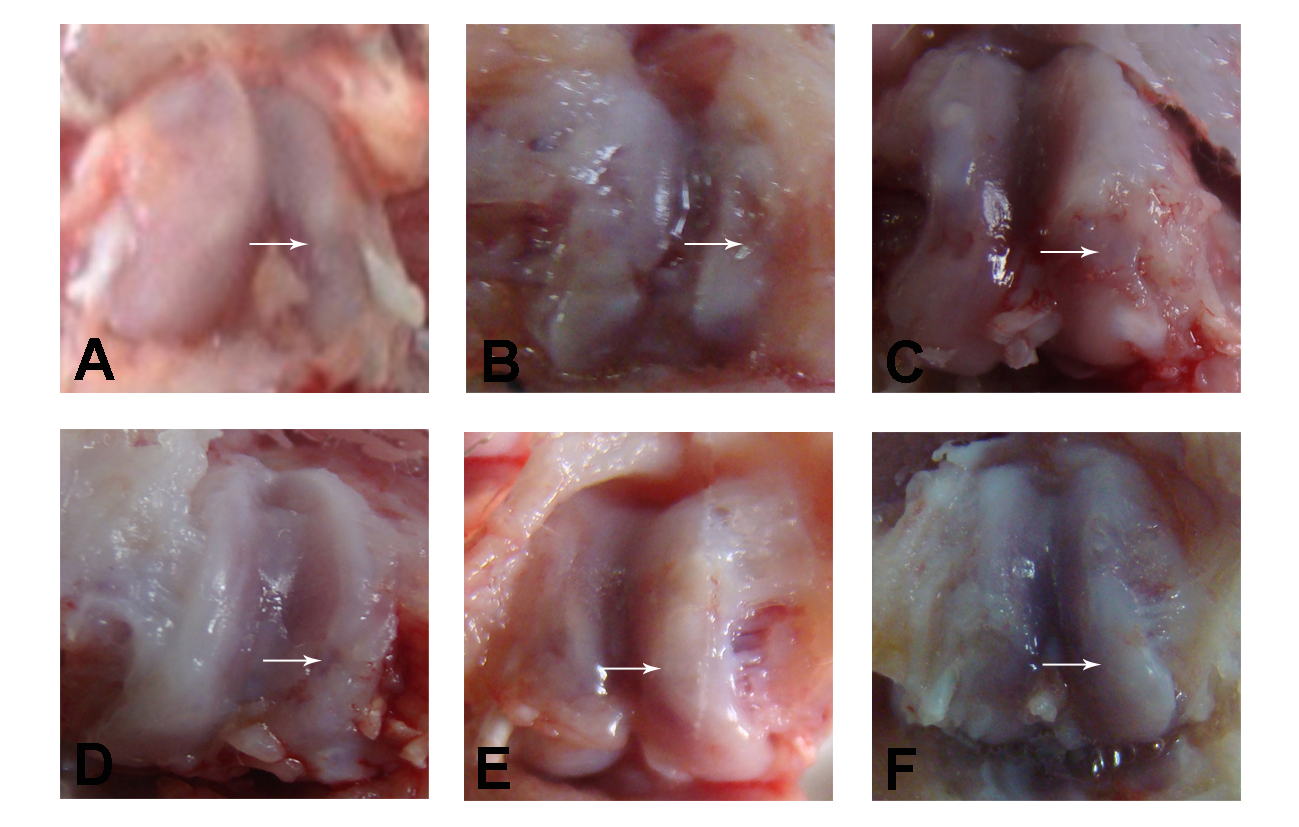

Supplement: Figure S2 — The gross anatomical change in the cartilage damage in a rat OA model injected by berberine. (A)Sham-operated group. (B) OAinduction group. (C) OA+the vehicle (water) group. (D) OA+Berberine (Low-dose) group. (E)OA+Berberine (Middle-dose). (F) OA+Berberine (High-dose). [file jcmm0018-0283-sd2.tif]

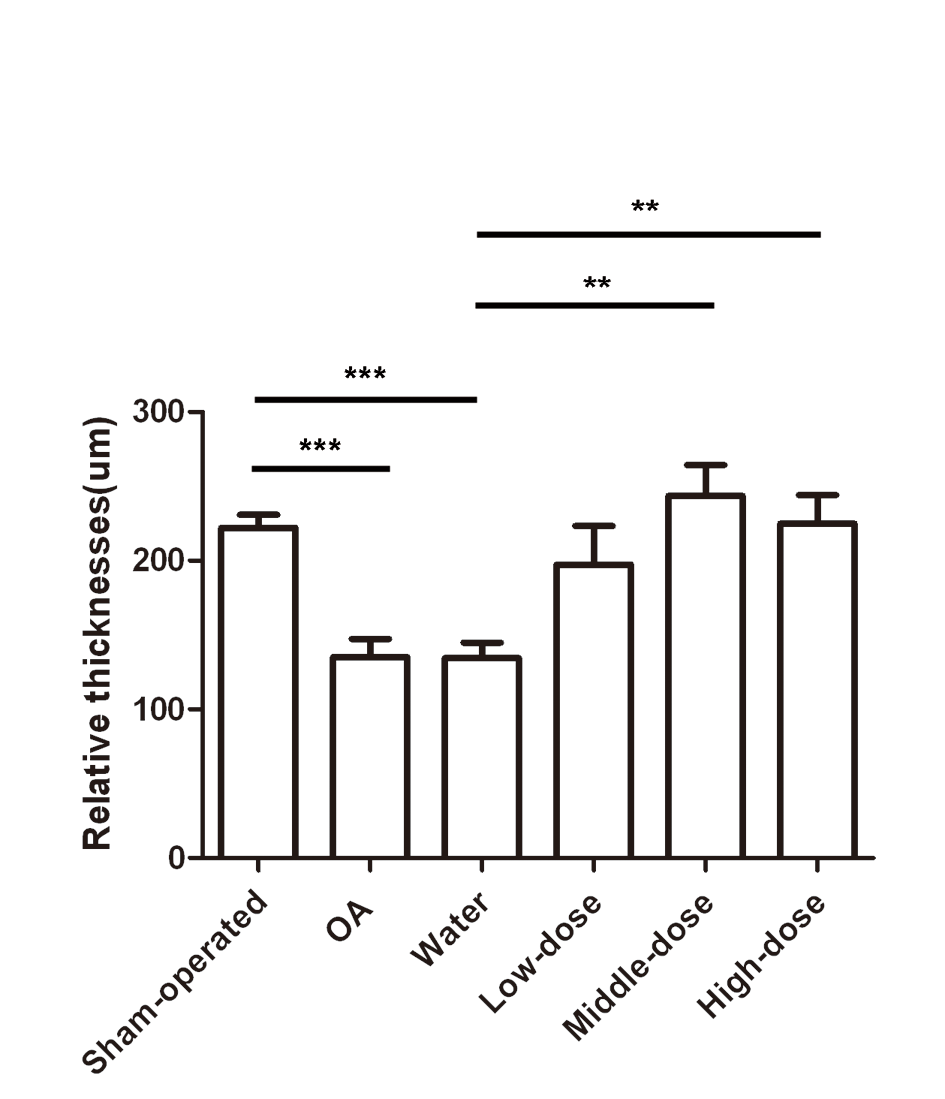

Supplement: Figure S3 — Histogram of the relative thickness of articular cartilage in a rat OA model injected by berberine. [file jcmm0018-0283-sd3.tif]
